# Antimicrobial Resistance Surveillance Methods in Bangladesh: Present and Way Forward

**DOI:** 10.1093/cid/ciad561

**Published:** 2023-12-20

**Authors:** Mohammad Julhas Sujan, Zakir Hossain Habib, Aninda Rahman, S M Shahriar Rizvi, Hridika Talukder Barua, Ahmed Taha Aboushady, Md Abul Hasnat, Saima Binte Golam Rasul, Hea Sun Joh, Kristi Prifti, Kyu-young Kevin Chi, Soo Young Kwon, Adam Clark, Sanjay Gautam, Marianne Holm, Florian Marks, John Stelling, Alina Shaw, Nimesh Poudyal

**Affiliations:** International Vaccine Institute, Seoul, Republic of Korea; Department of Microbiology, Institute of Epidemiology, Disease Control and Research (IEDCR), Directorate General of Health Services, Ministry of Health and Family Welfare (MoHFW), Bangladesh; Communicable Disease Control, Directorate General of Health Services, Ministry of Health and Family Welfare, Dhaka, Bangladesh; Communicable Disease Control, Directorate General of Health Services, Ministry of Health and Family Welfare, Dhaka, Bangladesh; International Vaccine Institute, Seoul, Republic of Korea; International Vaccine Institute, Seoul, Republic of Korea; Brigham & Women's Hospital, Harvard Medical School, Boston, Massachusetts, USA; International Vaccine Institute, Seoul, Republic of Korea; Department of Microbiology, Institute of Epidemiology, Disease Control and Research (IEDCR), Directorate General of Health Services, Ministry of Health and Family Welfare (MoHFW), Bangladesh; International Vaccine Institute, Seoul, Republic of Korea; International Vaccine Institute, Seoul, Republic of Korea; International Vaccine Institute, Seoul, Republic of Korea; International Vaccine Institute, Seoul, Republic of Korea; Brigham & Women's Hospital, Harvard Medical School, Boston, Massachusetts, USA; International Vaccine Institute, Seoul, Republic of Korea; Research & Collaboration, Anka Analytica, Melbourne, Australia; International Vaccine Institute, Seoul, Republic of Korea; International Vaccine Institute, Seoul, Republic of Korea; Cambridge Institute of Therapeutic Immunology and Infectious Disease, University of Cambridge School of Clinical Medicine, Cambridge, United Kingdom; Heidelberg Institute of Global Health, University of Heidelberg, Heidelberg, Germany; Madagascar Institute for Vaccine Research, University of Antananarivo, Antananarivo, Madagascar; Brigham & Women's Hospital, Harvard Medical School, Boston, Massachusetts, USA; Public Health Surveillance Group, LLC, Princeton, New Jersey, USA; International Vaccine Institute, Seoul, Republic of Korea

**Keywords:** active surveillance, case-based surveillance, laboratory-based surveillance, AMR surveillance, Bangladesh

## Abstract

The Institute of Epidemiology, Disease Control and Research (IEDCR) conducts active, case-based national antimicrobial resistance (AMR) surveillance in Bangladesh. The Capturing Data on Antimicrobial Resistance Patterns and Trends in Use in Regions of Asia (CAPTURA) project accessed aggregated retrospective data from non-IEDCR study sites and 9 IEDCR sites to understand the pattern and extent of AMR and to use analyzed data to guide ongoing and future national AMR surveillance in both public and private laboratories. Record-keeping practices, data completeness, quality control, and antimicrobial susceptibility test practices were investigated in all laboratories participating in case-based IEDCR surveillance and laboratory-based CAPTURA sites. All 9 IEDCR laboratories recorded detailed case-based data (n = 16 816) in electronic format for a priority subset of processed laboratory samples. In contrast, most CAPTURA sites (n = 18/33 [54.5%]) used handwritten registers to store data. The CAPTURA sites were characterized by fewer recorded variables (such as patient demographics, clinical history, and laboratory findings) with 1 020 197 individual data, less integration of patient records with the laboratory information system, and nonuniform practice of data recording; however, data were collected from all available clinical samples. The analyses conducted on AMR data collected by IEDCR and CAPTURA in Bangladesh provide current data collection status and highlight opportunities to improve ongoing data collection to strengthen current AMR surveillance system initiatives. We recommend a tailored approach to conduct AMR surveillance in high-burden, resource-limited settings.

## Teaser

In this study we collected and explored retrospective antimicrobial resistance data from microbiology laboratories across Bangladesh and compared with those from active case-based surveillance carried out by the national surveillance network to inform policy.

The spread of antimicrobial-resistant (AMR) bacteria is a significant global public health threat [1]. The misuse of antibiotics in humans, animals, and the environment and a “largely unregulated pluralistic health system” aggravate the situation, especially in low and middle-income countries (LMICs) in South Asia [2, 3]. In Bangladesh, for example, antibiotics are readily available over-the-counter in pharmacies, with up to 59.4% of nonprescribed antibiotics dispensed from the World Health Organization's (WHO) Access group, 46.5% from the Watch group, and 44% from the Reserve group [4]. The Communicable Disease Control (CDC) program is the National Action Plan's (NAP) implementing agency for the containment of AMR, and AMR surveillance data play a vital role in this regard. Strengthening and gradually expanding this system is an important priority [5]. Although the CDC programme was established to implement the NAP to address AMR containment in Bangladesh from 2017 to 2022 [6], the availability of surveillance data remains a challenge for accurately assessing the actual burden of AMR in the country [1, 7].

**Figure 1.**
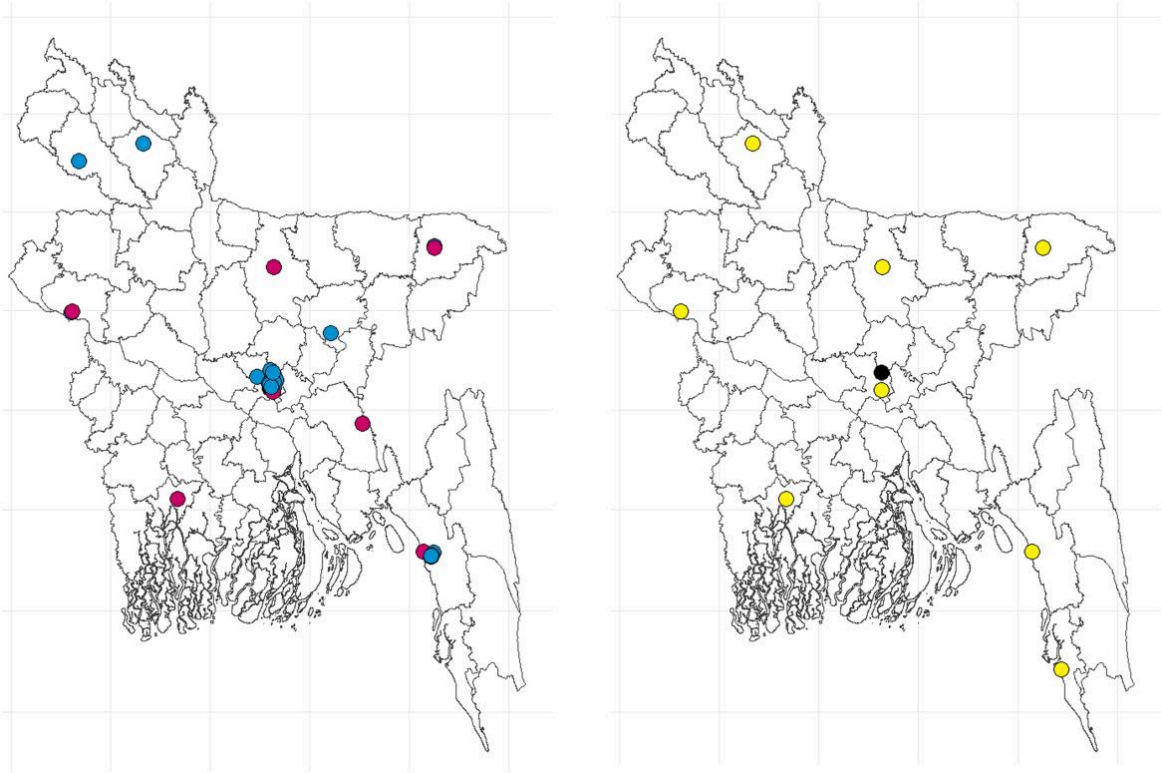
Capturing Data on Antimicrobial Resistance Patterns and Trends in Use in Regions of Asia (CAPTURA) and Institute of Epidemiology, Disease Control and Research (IEDCR) participating laboratories across Bangladesh. Magenta dots represent CAPTURA public laboratories, cyan dots represent CAPTURA private laboratories, yellow represents IEDCR public laboratories, and black represents IEDCR private laboratory.

The CDC and the Institute of Epidemiology, Disease Control and Research (IEDCR) have led AMR activities since the release of Bangladesh's NAP in 2017 [6]. The IEDCR is engaged in AMR surveillance activities in 8 public hospitals (Figure 1*B*), which are primarily located in urban settings. It has now been expanded to 11 sites. With a web platform [5] linked to the WHO Global Antimicrobial Resistance and Use Surveillance System (GLASS), IEDCR provides real-time access to sample case information recorded in each sentinel site, including information on patient demographics, clinical data, and microbiological culture data [8]. While the IEDCR represents the sectoral AMR Surveillance Coordination Centre and the National Reference Laboratory for national AMR surveillance activities in Bangladesh, other stakeholders, such as the Directorate General of Drug Administration, the Department of Livestock Services, the WHO, the Development Alternatives Incorporated (DAI), Fleming Fund Country Grant, the Medicines, Technologies and Pharmaceutical Services program (MTaPS), and the Capturing Data on Antimicrobial Resistance Patterns and Trends in Use in Regions of Asia project (CAPTURA, a regional Fleming Fund initiative), are actively engaged in improving antimicrobial resistance, consumption, and use surveillance activities in the country [9].

CAPTURA initiated its engagement in Bangladesh in November 2019 with stakeholder interactions and planning of program implementation. With commitment from and coordination with the CDC and IEDCR, CAPTURA was formally initiated in the country in May 2020 during the peak of the coronavirus disease 2019 pandemic. One of the major aims of CAPTURA was to explore available retrospective data to conduct laboratory-based AMR surveillance in Bangladesh. CAPTURA also aimed to manifest the challenges and limitations of the existing AMR surveillance system by assessing laboratory conditions using the Rapid Laboratory Quality Assessment tool [10]. This article describes the CAPTURA project activities of identifying data sources, data collection, record-keeping practices, data completeness, antimicrobial susceptibility testing (AST) practices, quality control, AST validity checks, and antimicrobial patterns and compares with the IEDCR-led active surveillance sites. These findings contribute to expanding AMR surveillance activities in the country.

## METHODS

### Study Design and Settings

We collected retrospective data on AST from 34 microbiology laboratories in Bangladesh between 2019 and 2022. Data were obtained from 10 public and 24 private laboratories (Figure 1*A*) and a comparative study was done to understand data completeness, quality control, data validation approaches, and patterns between IEDCR and CAPTURA.

### Data Collection

#### IEDCR

Based on the preliminary analysis plan developed by the CAPTURA team, a list of the necessary variables was shared with the IEDCR technical team in order to export the records from 2017 to 2019 into Microsoft Excel. Records on the IEDCR surveillance system were extracted from the surveillance database available through the web link (https://dashboard.iedcr.gov.bd/amr/). We received 16 816 individual isolate records on AST. The IEDCR surveillance platform stored records from 9 sentinel sites, 8 of which were public and 1 private. The data were converted into the WHONET format using the WHONET BacLink program [11]. Furthermore, patient personal identifiers were encrypted by using the WHONET encryption function to ensure patient anonymity and with accessibility limited to essential personnel.

#### CAPTURA

**Figure 2.**
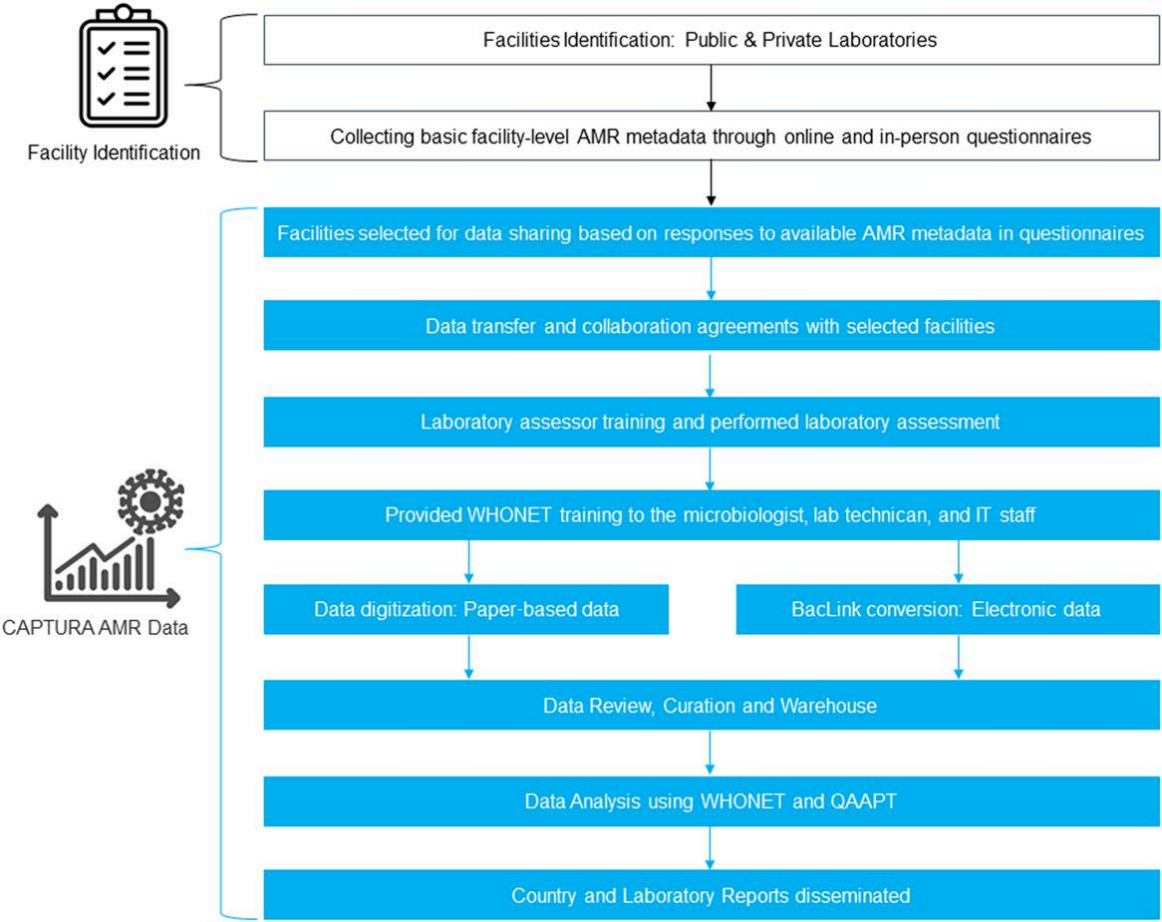
Capturing Data on Antimicrobial Resistance Patterns and Trends in Use in Regions of Asia (CAPTURA) approach to facility identification, training, data collection, analysis, and result dissemination. Abbreviations: AMR, antimicrobial resistance; CAPTURA, Capturing Data on Antimicrobial Resistance Patterns and Trends in Use in Regions of Asia; IT, information technology; QAAPT, Quick Analysis of Antimicrobial Patterns and Trends.

CAPTURA conducted 32 WHONET training sessions (on-site and virtual) as a capacity-building activity in the participating laboratories (Figure 2). Laboratory staff at CDC, IEDCR, and the surveillance sites were trained to use WHONET for data digitization and analysis. In addition, at least 1 team per laboratory was trained to record and share the data with CAPTURA via a digital window. A specific list of data variables guided AST data extraction for downstream analysis. As with IEDCR, CAPTURA collaborated with the software development team to provide technical support and fund those settings with electronic data management systems to enhance their capacity for digital data transfer.

The data collection process was followed by the creation of a metadata readme file with information on patient demographics, hospital identification details, laboratory procedures, and results from each site (Supplementary Table 1). This allowed for distinguishing data between study sites and data quality monitoring.

Paper-based information was manually entered to the WHONET system by laboratory personnel previously trained by the CAPTURA team. Similarly, to export electronic records into Excel format, the CAPTURA team provided technical and financial assistance to the participating laboratories to develop a function within the system.

### Data Validation

Once the data were transferred from sites to the CAPTURA secure cloud data warehouse [12, 13], several data cleaning stages were conducted, including the removal of redundant entries. This was followed by eliminating outliers such as inconsistent date formats, incorrect nomenclatures, and identifying data entry errors using WHONET software quality functions. Since each dataset was obtained from a different data management system, a tailored approach for data curation was required. In addition to data cleaning using WHONET, a manual examination was performed to curate the information. Additional SQLite Database Browser [14] software was used to detect outliers such as naming the microorganisms incorrectly, missing records on specimen type and dates, and inaccurate and inconsistent AST results. Afterward, the different datasets were combined using the WHONET data merge and encryption tool for further analysis.

### Data Analysis

The CAPTURA project collaborated with the WHONET development team to develop specific analysis capabilities that could also be of value in the context of other countries. WHONET was used to analyze the epidemiological information and quality control of microbiological tests, such as patient and sample statistics, microorganism and antibiotic statistics, isolate alerts, presence of multidrug resistance, GLASS results, cluster detection, data validity, quality control testing, capacity for organism detection, AST best practices, and quality control alerts. Similarly, the CAPTURA team developed the Quick Analysis of Antimicrobial Patterns and Trends (QAAPT) [15] tool to expand these analysis and add web-based visualization for patterns and trends within the AST results.

### Project Approval

The CAPTURA consortium project received an official approval from the Bangladesh CDC, Directorate General of Health Services (DGHS), Ministry of Health and Family Welfare, on 17 May 2020 (reference number DGHS/DC/ARC/2020/1708). Prior to data collection, a tri-party agreement was made between the DGHS, corresponding facility, and CAPTURA.

## RESULTS

###  

#### Data Variables Availability and Quality in IEDCR and CAPTURA Sites

In CAPTURA-led study, a total of 1 020 197 individual records from 33 hospitals/laboratories were collected. This included data recorded in handwritten registers, electronic formats and, in some sites, or a combination of both (Figure 1). IEDCR surveillance recorded data (n = 16 816) with multiple variables, such as patient general information (n = 6), demographic information (n = 10), clinical information (n = 5), previous antibiotic treatment history (n = 6), comorbidity (n = 6), provisional diagnosis history (n = 5), specimen information (n = 6), microorganism isolated, and AST results with different antibiotics with zone of inhibition (Supplementary Table 2). These data were documented by dedicated personnel for patients attending inpatient and outpatient departments.

In CAPTURA sites with handwritten data entry systems (n = 18 sites), variables such as patient encrypted ID, age, sex, clinical department, ward, unit, specimen number, specimen date, specimen type, microorganism reported, and AST results were collected from the patient registry in the microbiology laboratory. Although 6 of 18 paper-based recording systems sites had laboratory information systems, data could not be retrieved primarily due to poor entry and management practices. For example, the data backup process was unreliable, and records were deleted every 6 months with some laboratories storing data in a single column in Microsoft Excel and use of Microsoft Word or a bioMérieux VITEK 2 database for backup. Similarly, in 12 hospital laboratories where data were stored in electronic systems, CAPTURA was able to extract evidence more effectively due to the storage of integrated information (including from patient admission to discharge). As a result, the data quality generated from these sites was comparable to IEDCR. However, there were differences in the numbers of antibiotics tested, microorganisms isolated, and specimen type across the 12 hospitals, with some missing department, unit, and other information. This impacted data quality during WHONET BacLink conversion. In laboratories with both data recording (paper-based and electronic) systems in place, records such as patient ID, age, sex, clinical data, ward, unit, specimen number, specimen date, specimen type, organism name, and AST findings were obtained from both sources and merged.

### Data Completeness in IEDCR and CAPTURA Sites

All of the laboratories participating in IEDCR surveillance recorded case-based data (patient demography, clinical history including antibiotic use, comorbidities, diagnosis, specimen details, and AST results) in electronic format. CAPTURA obtained data from 34 sites. Among 33 of the CAPTURA sites, 18 had paper-based registers, 12 had electronic systems, and 3 had both formats. Additionally, the 34th dataset represented the aggregate available data from all 9 IEDCR sites.

Data completeness was calculated for each dataset based on the GLASS 8 high-priority variable [16]. While electronic data keeping in the IEDCR sites was 100% complete for all variables, the CAPTURA sites with similar systems recorded more incomplete data for 5 areas, such as department and ward. While the paper-based registers had less complete data across 8 fields, the CAPTURA sites with a combination of paper-based and electronic systems were as complete as the IEDCR sites (Table 1).

**Table 1.** Data Completeness and Quality Metrics

Variables	IEDCR Sites^a^	CAPTURA Sites		
	Electronic Entry (n = 7)	Paper-Based Entry (n = 18)	Electronic Entry (n = 12)	Paper-Based and Electronic Entry (n = 3)
Identification number	100%	99%	100%	100%
Age	100%	99.6%	99.7%	100%
Microorganism	100%	100%	99.8%	100%
Sex	100%	76%	100%	100%
Specimen type	100%	99.7%	99.9%	100%
Specimen date	100%	99.6%	100%	100%
Department	100%	86%	96%	100%
Location type	100%	98%	88%	100%
Antibiotics tested, No.^b^	36	99	85	68

Abbreviations: CAPTURA, Capturing Data on Antimicrobial Resistance Patterns and Trends in Use in Regions of Asia; IEDCR, Institute of Epidemiology, Disease Control and Research.

^a^All IEDCR sites used an electronic data recording system.

^b^Number of antibiotics tested at IEDCR and CAPTURA sites. Since all IEDCR sites used a uniform antibiotic panel in accordance with standard operating procedures, fewer antibiotics were tested at IEDCR sites than at CAPTURA sites.

### Microorganisms Reported in IEDCR and CAPTURA Sites

Ten bacterial species were commonly reported in IEDCR sites, including *Escherichia coli*, *Pseudomonas aeruginosa*, *Klebsiella pneumoniae*, *Staphylococcus aureus*, *Proteus* sp, *Acinetobacter baumannii* complex, *Salmonella* Typhi, *Enterococcus* sp, *Streptococcus pneumoniae*, *Vibrio cholerae*, *and Shigella* sp. In CAPTURA sites, most of the microorganisms were reported at the genus level and recording format was inconsistent; for example, bacterial nomenclatures in handwritten registers were different electronic platforms (Table 2).

**Table 2.** Commonly Reported Microorganisms in Surveillance Sites

IEDCR Sites^a^	CAPTURA Sites		
	Paper-Based Entry	Electronic Entry	Paper-Based and Electronic Entry
*Acinetobacter baumannii* complex	*Enterococcus* sp	*Acinetobacter* sp	*Acinetobacter* sp
*Enterococcus* sp	*Escherichia coli*	*Enterococcus faecalis*	*Enterococcus* sp
*Escherichia coli*	*Klebsiella pneumoniae*	*Enterococcus* sp	*Escherichia coli*
*Klebsiella pneumoniae*	*Klebsiella* sp	*Escherichia coli*	*Klebsiella* sp
*Proteus* sp	*Proteus* sp	*Klebsiella pneumoniae*	*Proteus* sp
*Pseudomonas aeruginosa*	*Pseudomonas aeruginosa*	*Klebsiella* sp	*Pseudomonas* sp
*Salmonella* Typhi	*Pseudomonas* sp	*Proteus* sp	*Salmonella* Typhi
*Vibrio cholerae*	*Salmonella* Typhi	*Pseudomonas* sp	*Staphylococcus aureus*
*Staphylococcus aureus*	*Staphylococcus aureus*	*Salmonella* Typhi	*Staphylococcus*, *coagulase* negative
*Shigella* sp	*Staphylococcus* sp	*Staphylococcus aureus*	*Streptococcus*, β-hemolytic

Abbreviations: CAPTURA, Capturing Data on Antimicrobial Resistance Patterns and Trends in Use in Regions of Asia; IEDCR, Institute of Epidemiology, Disease Control and Research.

^a^All IEDCR sites used an electronic data recording system.

### Invalid AST Reports in IEDCR and CAPTURA Sites

We analyzed the number of invalid AST reports as a proxy in order to study the quality standards of AST in IEDCR and CAPTURA sites. An invalid AST was described when the testing antibiotic was inconsistent with Clinical and Laboratory Standards Institute (CLSI) AST standards M100 [17] such as the testing of antibiotics for which no quality control or interpretative criteria have been defined. As representatives of gram-positive and gram-negative bacteria, antibiotic selection for testing practices for *S. aureus* and *E. coli* was examined (Tables 3 and 4). CAPTURA sites provided 60 times more records than IEDCR sites total (n = 1 020 197 vs n = 16 816, respectively) since IEDCR is limited to selecting only 6 specimens and reporting for 10 microorganisms.

**Table 3.** Proportion of Invalid Antimicrobial Susceptibility Test Results Recorded for *Staphylococcus aureus*

Antibiotics	IEDCR Sites^a^	CAPTURA Sites		
	Electronic Entry (n = 1057)	Paper-Based Entry (n = 15 255)	Electronic Entry (n = 41 231)	Paper-Based and Electronic Entry (n = 1283)
Amikacin/fosfomycin	NA	104 (0.7%)	NA	NA
Amoxicillin	NA	5255 (34.5%)	2033 (4.9%)	5 (0.4%)
Carbenicillin	NA	98 (0.6%)	NA	NA
Cefatrizine	NA	121 (0.8%)	NA	NA
Cephalexin	24 (2.3%)	15 (0.1%)	1322 (3.2%)	NA
Cephradine	NA	2335 (15.3%)	4360 (10.6%)	27 (2.1%)
Cloxacillin	NA	4639 (30.4%)	3904 (9.5%)	399 (31.1%)
Daptomycin	NA	21 (0.1%)	NA	NA
Fusidic acid	237 (22.4%)	98 (0.6%)	5668 (13.8%)	206 (16.1%)
Kanamycin/cephalexin	NA	216 (1.4%)	3996 (9.7%)	NA
Novobiocin	NA	50 (0.3%)	55 (0.1%)	NA
Oxacillin	171 (16.2%)	506 (3.3%)	5543 (13.4%)	NA
Pefloxacin	NA	137 (0.9%)	NA	NA
Penicillin V	308 (29.1%)	268 (1.8%)	1150 (2.8%)	NA
Tazobactam	NA	48 (0.3%)	NA	92 (7.2%)
Tigecycline	NA	80 (0.5%)	1618 (3.9%)	88 (6.9%)
Vancomycin	317 (30.0%)	1264 (8.3%)	11 582 (28.1%)	466 (36.3%)

Data are presented as No. (%).

Abbreviations: CAPTURA, Capturing Data on Antimicrobial Resistance Patterns and Trends in Use in Regions of Asia; IEDCR, Institute of Epidemiology, Disease Control and Research; NA, not available.

^a^All IEDCR sites used an electronic data recording system.

**Table 4.** Proportion of Invalid Antimicrobial Susceptibility Test Results Recorded for *Escherichia coli*

Antibiotics	IEDCR Sites^a^	CAPTURA Sites		
	Electronic Entry (n = 940)	Paper-Based Entry (n = 18 995)	Electronic Entry (n = 38 005)	Paper-Based and Electronic Entry (n = 1427)
Amikacin/fosfomycin	NA	1085 (5.7%)	NA	NA
Amoxicillin	NA	8197 (43.2%)	11 174 (29.4%)	5 (0.4%)
Carbenicillin	NA	1912 (10.1%)	NA	NA
Cefotaxime	NA	NA	NA	497 (34.83%)
Cefatrizine	NA	726 (3.8%)	NA	NA
Cefepime	NA	238 (1.3%)	NA	NA
Cefoperazone/sulbactam	NA	5 (0.03%)	3230 (8.5%)	NA
Cephalexin	769 (81.8%)	205 (1.1%)	3148 (8.3%)	NA
Cephradine	NA	3189 (16.8%)	11 458 (30.2%)	91 (6.4%)
Cloxacillin	NA	243 (1.9%)	453 (1.2%)	NA
Fusidic acid	46 (4.9%)	202 (1.1%)	NA	NA
Kanamycin/cephalexin	NA	412 (2.2%)	1905 (5.0%)	14 (1.0%)
Oxacillin	17 (1.8%)	662 (3.5%)	NA	NA
Pefloxacin	NA	59 (0.3%)	NA	NA
Penicillin V	42 (4.5%)	62 (0.3%)	NA	NA
Tazobactam	NA	22 (0.1%)	6419 (16.9%)	498 (34.9%)
Tigecycline	NA	359 (1.9%)	218 (0.6%)	319 (22.4%)
Vancomycin	66 (7.0%)	1417 (7.5%)	NA	3 (0.2%)

Data are presented as No. (%). The total number of records from IEDCR electronic entry #16 816, CAPTURA paper-based entry #338 142, CAPTURA electronic entry #640 904, and CAPTURA both electronic and paper entry #41 151.

Abbreviations: CAPTURA, Capturing Data on Antimicrobial Resistance Patterns and Trends in Use in Regions of Asia; IEDCR, Institute of Epidemiology, Disease Control and Research; NA, not available.

^a^All IEDCR sites used an electronic data recording system.

For *S. aureus*, invalid test records were available for 5 antibiotics in IEDCR surveillance, while CAPTURA extracted data for 17, 11, and 7 drugs in paper-based, electronic, and combination entries, respectively (Table 3). Overall, the IEDCR and CAPTURA electronic records had a comparable proportion of invalid AST recorded (6.28% and 6.43%, respectively). For individual antibiotics, a higher proportion of invalid tests was found in IEDCR for fusidic acid and oxacillin, compared to CAPTURA.

Among CAPTURA sites, the proportion of invalid tests was higher in electronic format (6.43%) than in paper-based records (4.51%), followed by combination entries (3.12%). A higher proportion of invalid tests was recorded in paper-based registers for amoxicillin and cephradine and 4 drugs in combination format (cloxacillin, fusidic acid, tigecycline, and vancomycin). Although the paper-based form had records for all 17 of 17 antibiotics, the proportion of invalid tests noted was lower than in other formats.

For *E. coli*, 5.6% (940/16 830) of records were categorized as invalid AST in IEDCR surveillance. WHONET defined the invalid test based on testing an antibiotic for which there were no testing guidelines. For CAPTURA, in comparison, this was 5.6% (18 995/338 142) in handwritten entries and 5.9% (38 005/640 904) and 3.5% (1427/41 151) for data extracted in electronic and combination formats, respectively. Like *S. aureus*, we observed 5 antibiotics included in IEDCR surveillance, while paper-based entry in CAPTURA had the highest number of antibiotics (17/18). Although different laboratories performed AST using a variety of antibiotics, the average for CAPTURA sites was 18. The most commonly recorded invalid test in IEDCR sites was for cephalexin, with approximately 82% of results not complying with CLSI guidelines. Similarly, for CAPTURA sites, invalid tests varied among recording formats, for example, amoxicillin (8197/18 995 [43.2%]) in paper-based entry, cephradine (11 458/38 005 [30.2%]) in electronic records, and tazobactam (498/1427 [34.9%]) only in sites with a combination record entry system. The paper-based data from CAPTURA sites included the greater numbers of antibiotics because different sites used different antibiotic panels. However, the proportion of invalid tests recorded for individual antibiotics was lower compared to IEDCR and CAPTURA sites with electronic recording systems. Notably, the first released standard operating procedure (SOP) for all IEDCR sites was corrected at the end of 2019, and CAPTURA collected data between 2017 and 2019.

**Figure 3.**
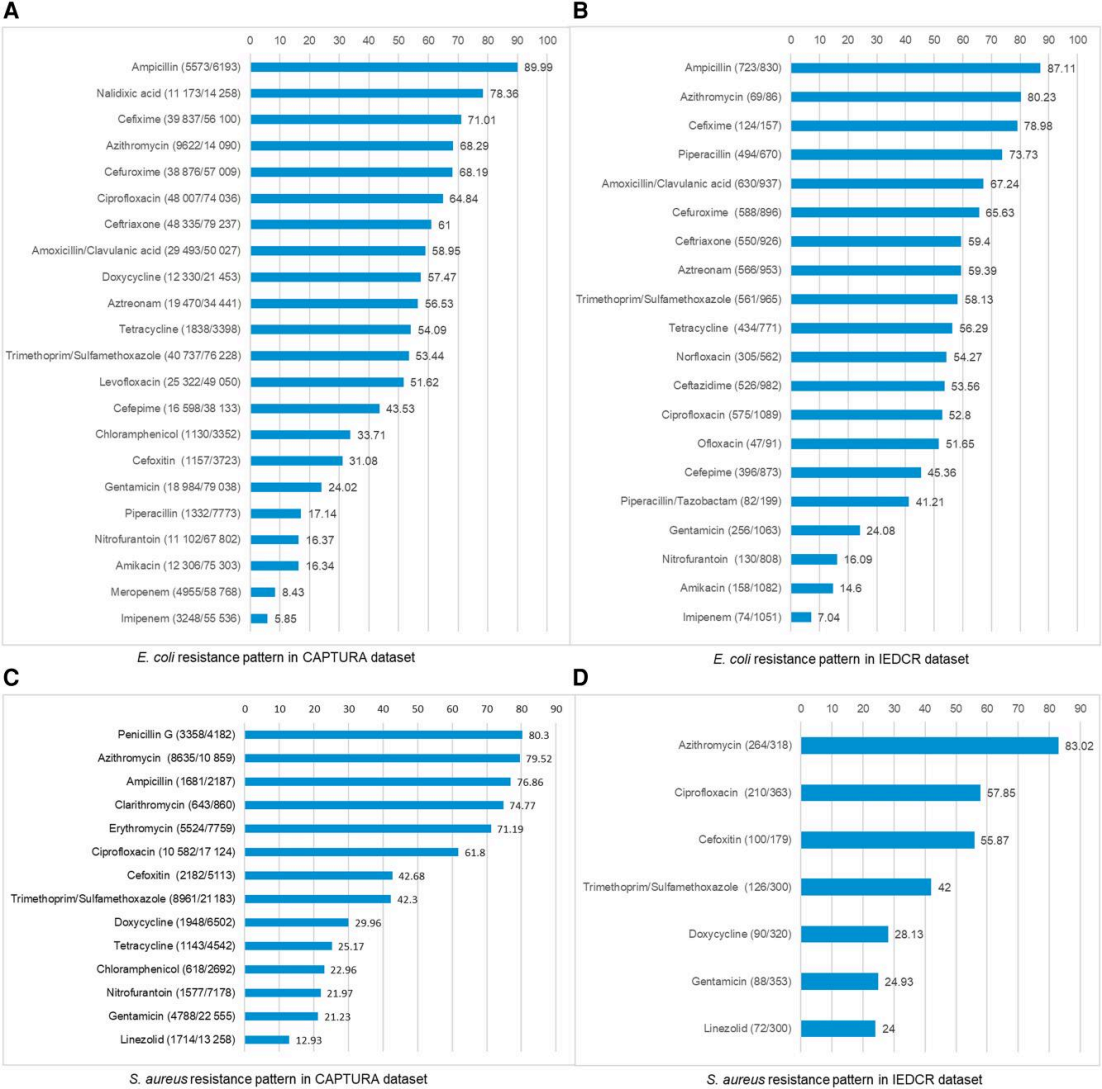
*A*, Patterns of *Escherichia coli* in the Capturing Data on Antimicrobial Resistance Patterns and Trends in Use in Regions of Asia (CAPTURA) dataset. *B*, *Escherichia coli* in the Institute of Epidemiology, Disease Control and Research (IEDCR) dataset. *C*, *Staphylococcus aureus* in the CAPTURA dataset. *D*, *Staphylococcus aureus* in the IEDCR dataset.

To understand the AMR pattern, we compared antibiotic resistance profiles for *E. coli* as a representative of gram-negative organisms and *S. aureus* as a representative of gram-positive organisms between the CAPTURA and IEDCR datasets. Approximately 90% of *E. coli* isolates were resistant to ampicillin whereas amikacin and imipenem were the effective drugs of choice in both datasets (Figure 3). For *S. aureus*, among the common tested drugs between IEDCR and CAPTURA sites, azithromycin was found to be most resistant whereas linezolid and gentamicin were susceptible drugs in both datasets (Figure 3). We observed comparable patterns in both datasets, which reflects AMR trends. For both isolates, there are difference in the antibiotic panels between IEDCR and CAPTURA sites (Figure 3). IEDCR sites have their own SOP according to the CLSI guideline and they follow the uniform antibiotic panels as instructed by the IEDCR. In contrary, CAPTURA sites were individual laboratories, and they followed their own manuals. As a result, there are similar antibiotics from the same group of drugs in the dataset of CAPTURA sites.

## DISCUSSION

In this study, we analyzed AMR surveillance data collected by IEDCR and CAPTURA sites in Bangladesh. IEDCR data were collected between 2017 and 2019, and CAPTURA data cover information from 2016 to 2020. Both IEDCR and CAPTURA sites were located primarily in urban areas. IEDCR's active surveillance collected patient information daily with at least 1 staff member stationed at each site. In contrast, CAPTURA accessed aggregated retrospective data from all non-IEDCR study sites input locally by laboratory staff as well as results from 9 case-based IEDCR sites.

The GLASS protocol highlights the importance of AMR data validity, consistency, and completeness [16]. Data completeness is key to AMR surveillance programs as the unavailability of complete quality information results in inaccurate reporting of AMR rates, which impacts the design and delivery of control measures [18]. The retrospective data available at CAPTURA sites were characterized by fewer recorded variables, less complete data, poor integration of patient records with the laboratory information system, inconsistent and unreliable data recording practices, and data collected all at once. However, data volume and geographic coverage were much higher than in IEDCR sites. The GLASS procedure, meanwhile, was followed in IEDCR sites, where records were only stored in an electronic database to define variability standards, resulting in better data completeness metrics. Detailed patient-level data were gathered from much smaller samples of isolates according to the data collection protocol. This difference is likely due to data sourcing methods; for example, CAPTURA collected retrospective data from all samples or all positive samples from sites routinely not participating in AMR surveillance, while IEDCR surveillance was designed to collect prospective case-based data by a trained individual in defined locations for a subset of all samples collected. Furthermore, the IEDCR approach provided more detailed and granular data for a small subset of samples processed in the laboratory. In contrast, the CAPTURA approach overlooked the clinical detail available within the IEDCR approach but incorporated available results from all samples or at least all positive samples. In both circumstances, differences in the availability of human and technical resources impacted the outcome. On-site IEDCR surveillance staff received more comprehensive training; followed a common laboratory SOP; were subject to external quality assurance and retesting procedures to maintain the quality of laboratory results; and had access to computers, software, and internet connectivity, all of which greatly impact the feasibility of AMR surveillance. Furthermore, we found that using electronic tools for record keeping was advantageous in both IEDCR and CAPTURA sites. For example, in most cases, paper-based registries complied poorly with GLASS procedures, lacked information on American Type Culture Collection and specimen details, and used an incorrect microbial nomenclature system. However, it should be noted that laboratory information systems from different providers were used in 9 of the 12 CAPTURA sites with electronic data recording systems, resulting in differences in defining and recording data variables. In addition, we found that 6 of 18 sites currently using a paper-based entry system had Laboratory Information System that were not in active use. Sanju et al (unpublished data) studied the challenges of implementing the WHONET/BacLink system in Nepal and reported the input of incomparable data elements across different software, inadequate routine training, frequent transfer of trained staff, poor real-time technical support, and integration issues with ongoing hospital information systems as key challenges to adopting such systems. With a challenge to sustainably implement technically demanding methods in LMICs, no such alternative should impact the collection of AMR data for ongoing system evaluation and capacity-building purposes in the long run.

Comparing the 10 most common microorganisms reported in IEDCR with the 3 different CAPTURA site recording systems demonstrates the difference in prevalence and species level classification of bacterial types. The WHO GLASS protocol lists pathogens for AMR surveillance [19]. Among the common pathogens identified in IEDCR and CAPTURA sites, approximately 50% were included in the GLASS priority list. This difference may be due to commonly circulating microorganisms in the community or inadequate laboratory capacity to report pathogens requiring more complex growth conditions, such as media supplements for *Neisseria gonorrhoeae* and *S. pneumoniae* [20]. Therefore, laboratories should be equipped to report pathogens with additional growth conditions while being able to characterize microorganisms at the species level. This is critical as it affects the selection, interpretation, and clinical correlation of AST results, impacting data quality and reliability for patient management and AMR surveillance.

Invalid AST results were analyzed as a proxy to understand the quality of microbiology laboratories in performing such tests. Invalid tests were defined when laboratory procedures were not consistent with the CLSI AST requirements M100 [17]. This included testing antimicrobials with no quality control or interpretation criteria available, incorrect bacteria–antibiotic combinations (eg, testing intrinsically resistant antibiotics), use of inaccurate antibiotic disk potency, technical errors with WHONET/BacLink configuration, differences in breakpoints suggested by the vendor and CLSI or European Committee on Antimicrobial Susceptibility Testing, and knowledge gaps regarding antibiotics used for characterization versus treatment (eg, novobiocin, ceftriaxone/clavulanic acid combination). Other factors include misinterpretation of surrogate testing results and the use of the disk diffusion method where minimum inhibitory concentration is recommended. There were fewer antibiotics with invalid AST for either *S. aureus* or *E. coli* (Tables 3 and 4) in IEDCR sites than in CAPTURA sites. This is likely related to the 36 antibiotics tested in IEDCR sites compared to at least 68 tested in CAPTURA sites, owing to the use of different antibiotic panels with distinct antibiotics. Additionally, if a disk is unavailable or if all the antibiotics in the panel are found to be resistant, the same facility may occasionally use an alternative antibiotic, sometimes on the request of physicians for the purpose of treatment. On the other hand, IEDCR sites only utilized the antibiotics mentioned in their SOP and typically did not experience a disk insufficiency problem. Compared to the electronic format, handwritten registers had a more significant number of antibiotics included for an invalid result. However, in the majority of cases, the proportion of unacceptable findings was higher in electronic format. In addition, we found that higher numbers of privately owned facilities record antibiogram data in electronic format compared to public hospitals/laboratories. This shows the advantages and disadvantages of each record-keeping and surveillance system. Although electronic data keeping and active case-based surveillance result in more intrinsically reliable data, human, and technical resource constraints limit the application of such methods in LMICs such as Bangladesh. Based on this study, we recommend a cautious approach with a gradual transition to advanced AMR surveillance practices such as electronic record-keeping systems. With a challenge to sustainably implement technically demanding methods in LMICs, no such alternative should impact the collection of AMR data for ongoing system evaluation and capacity-building purposes in the long run.

One of the CAPTURA activities included facilitating the adoption of electronic AMR data collection methods in our study sites. We provided technical support to develop a function within existing systems to export patient and laboratory information and we developed an open-source data visualization tool, QAAPT [15], which is compatible with BacLink converted WHONET datasets and integrated with the District Health Information System (DHIS2) software [21]. The laboratory-based surveillance network can be expanded with the aid of such an interoperability strategy.

## CONCLUSIONS AND FUTURE PERSPECTIVE

**Figure 4.**
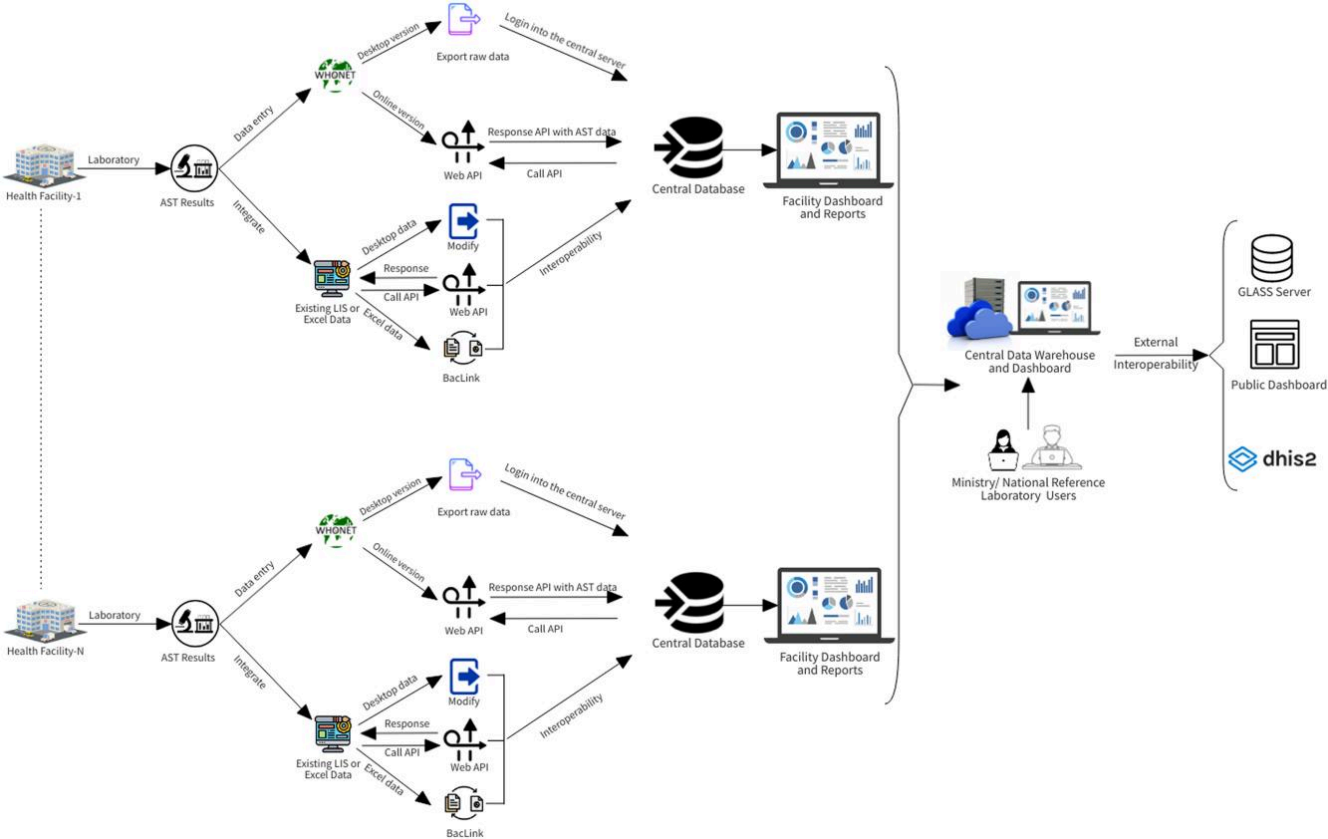
The proposed architecture of the antimicrobial resistance integrated surveillance system in Bangladesh. Abbreviations: API, Application Programming Interface; AST, antimicrobial susceptibility testing; GLASS, Global Antimicrobial Resistance and Use Surveillance System; LIS, Laboratory Information System.

While we found data quality and reliability differences between IEDCR and CAPTURA sites, this study demonstrates the feasibility of expanding the number of surveillance sites beyond those led by IEDCR by assessing the existence of quality AST methodologies and data collection practices for a more robust and geographically representative surveillance system. Considering the substantial resources required for case-based surveillance, it is possible to select more sites for laboratory-based surveillance on the same platform in order to perform quality control and improvement activities and to establish coordinated laboratory networking. Prioritizing the transition from current paper-based practices to electronic platforms will help to ensure data management quality; however, to ensure overall validity of microbiology data for surveillance of resistance will require extensive external quality assurance initiatives and proper assessments of potential selection biases in surveillance data. To improve existing processes and continue working toward controlling the increasing burden of AMR, we propose establishing an AMR integrated surveillance system in Bangladesh that will communicate information with unique laboratory or health information systems, WHONET software, and the existing IEDCR surveillance system (Figure 4). The platform could incorporate an advanced analysis pipeline with dashboards available for public and restricted use. In such a scenario, each laboratory would have access to the record and would edit and monitor their data, which could then be shared for policy purposes. In addition, this platform would allow laboratories to download a list of organisms, specimens, hospital departments, and antibiotics with standard names and codes, enabling uniform record-keeping practice. Beyond expanding AMR surveillance, this could also contribute to addressing the need for an integrated hospital information system, which is largely missing in public healthcare facilities in Bangladesh.

## Supplementary Data

Supplementary materials are available at *Clinical Infectious Diseases* online. Consisting of data provided by the authors to benefit the reader, the posted materials are not copyedited and are the sole responsibility of the authors, so questions or comments should be addressed to the corresponding author.

## Supplementary Material

ciad561_Supplementary_Data

## Notes

***Disclaimer.*** The views expressed here are those of the authors and not necessarily those of the UK Department of Health and Social Care or its management agent, Mott MacDonald; and, International Vaccine Institute.

***Acknowledgments.*** The authors express their gratitude to the Communicable Disease Control and Institute of Epidemiology, Disease Control and Research (IEDCR) directors, hospital administration, microbiologists, data entry operators, third-party software vendors, software developers from different hospitals, laboratory technologists, and others who assisted CAPTURA in digitizing the data.

***Supplement sponsorship.*** This article appears as part of the supplement “Capturing Data on Antimicrobial Resistance Patterns and Trends in Use in Regions of Asia (CAPTURA),” funded by the Department of Health and Social Care's Fleming Fund using UK aid.

***Financial support.*** This work was supported by the Fleming Fund regional grants (grant numbers FF10-135 and FF11-139). The Fleming Fund is a UK aid investment to support surveillance of antimicrobial resistance in low- and middle-income countries in Africa and Asia. The regional grants program is managed by Mott MacDonald Ltd, the Fleming Fund Grants Management Agent.
