# Supplementary material for: Antimicrobial Resistance Surveillance Methods in Bangladesh: Present and Way Forward
Source: Clin Infect Dis. 2023 Dec 20;77(Suppl 7):S549–59. doi: 10.1093/cid/ciad561 (PMC10732563; doi:10.1093/cid/ciad561)
Supplement: ciad561_Supplementary_Data [file ciad561_supplementary_data.docx]

**Supplementary Table 1.** List of variables included in the metadata readme file

| **Section** | **Items** | **Responses** |
| --- | --- | --- |
| **Details on File** | File name |  |
|  | *CAPTURA ID* | [IVI to fill out this section] |
|  | Facility Name |  |
|  | Type of data [AMR/U/C/c] | AMR |
|  | Name of person who uploaded dataset to Cloud and Date of upload (dd/mm/yyyy) |  |
|  | Name of person completing this template |  |
|  | Country |  |
|  | Remarks |  |
| **Data Description** | Number of data variables (columns) |  |
|  | Number of observations (rows) |  |
|  | Is a data dictionary available (Yes /No/ Don’t know)? |  |
|  | Is the dictionary uploaded in the warehouse (Yes /No/ Don’t know)? |  |
|  | Is there any other associated files/documentation uploaded in the warehouse (Yes /No/ Don’t know)?  If yes, please give brief description. |  |
|  | Time-period of dataset (e.g., month and year range)  If the dataset is made of multiple years, please specify the period for each year (e.g., 2016 – Jan to July,  2017 – Jan to Sept) |  |
|  | Geographic area of dataset  Please specify as much as possible (e.g., wards of hospital, district/town facility generally serves) |  |
|  | OTHER REMARKS |  |
| **AMR Data** | | |
| **Data Quality** | Is there any data duplicated across files? |  |
|  | Is the data complete (e.g., are all data included or just the first 10 per month)? |  |
|  | Are negative testing results included (e.g., no pathogen detected)? |  |
|  | Are a number of non-viable or destroyed samples available (e.g., samples which couldn’t be cultured or identified)?  If yes, please describe it as best to your knowledge. |  |
|  | What criteria were used to collect data (e.g., only blood samples, only inpatient)? |  |
|  | Were there any significant changes which may have affected the data available in each file?  E.g.,  - New equipment introduced (e.g., **VITEK® 2**)  - Quality control system introduced/enhanced  - Changes in guidelines used (e.g., CLSI to EUCAST)  - Changes in testing protocols (e.g., not testing urine) |  |
|  | Was there any testing for new antimicrobials (panels added/expanded)? |  |
|  | Was testing interrupted or changed during the data collection period  (for example, machine X was broken in June 2018 so we couldn’t test Y)? |  |
|  | Remarks |  |
|  | Please describe how you gathered the information provided above |  |
| **Data Denominator** | Population data for area | Sources of denominator: |
|  | Number of hospital/ward inpatient days (per day/month/year) | Sources of denominator: |
|  | Number of yearly admissions (per year) | Sources of denominator: |
|  | Number of hospitals outpatient visits (per day/month/year) | Sources of denominator: |
|  | Number of bacterial cultures processed (per day/month/year) | Sources of denominator: |
|  | Number of blood cultures processed (per day/month/year) | Sources of denominator: |
|  | [If you can get other similar and useful information not listed here, please include here] | Sources of denominator: |
| **CAPTURA Data** | Rapid Laboratory Quality Assessment (RLQA) score | [IVI to fill out this section] |

**Supplementary Table 2. List of Data Variables in IEDCR surveillance**

| General information | 1. Case ID 2. Interview date 3. Hospital registration number of the patient 4. Treatment received from 5. Department 6. IPD patient ward number |
| --- | --- |
| Patient personal profile | 1. Name 2. Age 3. Gender 4. Father/ Husband’s name 5. Mother’s name 6. Occupation 7. Educational status 8. Mailing address 9. Mobile number of the respondent 10. Name and mobile number of family member |
| Clinical Information | 1. Urinary Tract Infection 2. Diarrhoea 3. Lower Respiratory Tract Infection 4. Wound Infection 5. Septicemia |
| Antibiotic treatment history | 1. Treated with antibiotic status 2. Type of antibiotics prescribed 3. Antibiotic name 4. Number of days taken antibiotic 5. Number of doses of antibiotic taken per day 6. No of missed doses |
| Admission to other hospital | 1. Status 2. Hospital information 3. Date of admission 4. Date of discharge 5. Number of days previous hospitalisation |
| Comorbidity | 1. Diabetes 2. Chronic Kidney Disease 3. Chronic Liver Disease 4. CKD 5. Cancer 6. Other |
| Provisional Diagnosis | 1. UTI 2. Diarrhoea 3. Wound infection 4. Lower respiratory tract infection 5. Septicemia |
| Specimen collected | 1. Urine 2. Stool 3. Wound swab/ pus 4. Sputum 5. Blood 6. Endotracheal aspirate |
| Organisms | Identified organisms |
| Antimicrobial Susceptibility Testing Results | 1. Zone of Inhibition value 2. R/I/S |
